# Economic impact of intestinal failure from a patient perspective – A pilot study

**DOI:** 10.1016/j.intf.2025.100070

**Published:** 2025-06-30

**Authors:** Maddison Breen, Quiney Lin, Liz Beyer, Michelle Cunich, Sharon Carey

**Affiliations:** a Nutrition and Dietetics Department, Royal Prince Alfred Hospital, Sydney, Australia; b Faculty of Medicine and Health, Central Clinical School, The University of Sydney, Sydney, NSW 2050, Australia; c Department of Gastroenterology and Hepatology, Royal Prince Alfred Hospital, Sydney, Australia; d Charles Perkins Centre, Faculty of Medicine and Health, Sydney Medical School, Central Clinical School, Central Sydney (Patyegarang) Precinct, The University of Sydney, Sydney, NSW 2050, Australia; e Sydney Health Economics Collaborative, Sydney Local Health District, Sydney, NSW 2050, Australia; f Institute of Academic Surgery, Royal Prince Alfred Hospital, Sydney, NSW 2050, Australia

**Keywords:** Parenteral nutrition - home, Health economics, Reimbursement, Out-of-pocket expenses, Intestinal failure, Income, Patient survey

## Abstract

**Background:**

Intestinal Failure (IF) is a complex and life-long illness, requiring home parenteral and/or enteral nutrition, multiple medications, dietary changes and input from multiple healthcare specialists. The aim of this pilot study is to quantify the economic impact of IF from the patient perspective.

**Materials and methods:**

A tailored questionnaire was developed, piloted and administered to adult IF patients within a quaternary hospital in Sydney, Australia. The questionnaire collected health-related out-of-pocket expenses (OOPE) over the past 12-months. Data was analysed using descriptive statistics (median and range).

**Results:**

A total of 13 participants responded to the questionnaire. OOPE in a 12-month period was AUD $6099 (AUD $3170 - $45,744); this was 24.4 % of annual reported income. OOPE for participants in metropolitan regions were higher than those living in regional or rural regions (AUD $6538 versus AUD $6099, respectively). Participants with a motility disorder spent more on total OOPE than those without a motility disorder (AUD $7178 versus AUD $3905); with the majority of people living in metropolitan regions. OOPE for medications was the highest individual cost per year, AUD $2160 (AUD $820 - $6000). Average potential lost income was AUD $62,310 per annum.

**Conclusion:**

This pilot study shows the significant economic burden for people living with IF, including loss of income. Results warrant expansion to assess burden over a larger patient group. IF services need to be aware of the financial burden experienced by this patient group and ensure they are supported to access financial aid where eligible.

## 1 Introduction

Intestinal failure (IF) is defined as the gut’s inability to absorb nutrients, water, and/or electrolytes to function normally [1]. The most common underlying diseases that cause IF are short bowel syndrome, intestinal fistulae, small bowel mucosal disease, mechanical or pseudo-obstruction and motility disorder [1], [2], [3]. People with IF usually require home parenteral nutrition (HPN) to administer nutrients, water and/or electrolytes for growth and function, ongoing for many months to years. These patients may also require supplemental enteral nutrition, numerous medications and specialist medical support in addition to HPN. HPN is life saving and can both improve and hinder quality of life for these patients [4], [5]. Although the hope is to cease HPN and return to a enteral or oral diet without requiring intravenous support, patients with IF usually require HPN indefinitely. In Australia, IF is a recognised rare condition, with an estimated 150–200 adults living with IF and requiring HPN [6].

People with IF often develop complications that become increasingly difficult to manage as an outpatient, are costly and in many cases, life-threatening [5]. The most common complications are catheter-related such as infections or occlusions. Other complications can include parenteral nutrition associated liver disease (PNALD), metabolic bone disease and acute kidney injuries or chronic renal disease often on the background of dehydration [1], [2]. These complications often require lengthy readmission to hospital [3].

Patients diagnosed with IF are usually managed by specialist teams within tertiary hospitals, where management is often resource intensive, demanding the care of a wide range of specialist medical professionals, nursing and allied health clinicians [1], [2]. In Australia, healthcare is underpinned by a universal health insurance scheme (Medicare) and the hospital-directed costs related to both IF and HPN (including ancillaries) are absorbed by the healthcare system. Some additional healthcare related out-of-pocket expenses (OOPE) including medications, stoma care, specialist medical appointments, tests and examinations are often subsidised by the public health system or partially paid through private health insurance. However, there are often OOPE remaining for patients. Furthermore, with specialist multidisciplinary teams typically centralised to metropolitan areas of Australia, there is a further financial impact for those living in regional and rural parts of Australia. This can include additional travel costs, as well as possible costs related to geographical distance from large centres, for example higher cost of specialist foods and delivery costs. It has previously been reported that 15 % of health care expenses in Australia are from out-of-pocket costs that patients have had to absorb [7].

Costing studies to date have examined the significant financial health care burden of IF specifically [3], [8], [9]. Siu et al. [3] found that the median cost associated with both initial hospital HPN training admissions and subsequent re-admissions for complication management or treatment of the underlying disease were nearly double the reimbursement provided by the Australian government. In Europe and the United Kingdom, EUR €60,000 – EUR €85,0000 was spent yearly on IF patients requiring HPN [8], with a third of healthcare system costs also due to HPN complications associated with hospital stays of greater than 2 weeks [8]. Not only are researchers examining the current economic burden of managing IF and subsequent complications, but some have explored ways to save some of these costs in the long term. Paediatric simulations in the Netherlands [10] have shown a cost saving of nearly USD $500,000 by utilising intestinal rehabilitation allowing children to wean off HPN. Canovai et al. [11] assessed cost effectiveness of intestinal transplant, the most complex and costly solid organ transplant. Although initially patients requiring transplant incur a substantially higher financial investment, after 5 years the healthcare system was spending less on transplanted patients than those stable at home on HPN.

IF, associated disease and/or HPN related complications continues to be a high cost to the health system [9], [12] even when patients can live at home with HPN. It is further acknowledged in the literature that this cost would be much higher if patients did not have HPN, and had to remain as inpatients in the hospital [9]. However, there is very little research assessing the financial burden for patients living at home on IF, despite research indicating chronic disease contributes significant financial burden for patients [13], [14]. Arhip et al. [15] is the only study to date that has assessed personal costs for patients receiving HPN. Results showed personal and lack of productivity costs were low in comparison to overall HPN costs, but cost to patients was estimated at EUR €729.49 and EUR €256.39 for the year respectively [15]. The aim of this pilot study, therefore, is to investigate the type of direct costs related to IF, and the quantity of these costs, from the Australian patient perspective. The secondary aim is to estimate the loss of labour income (an important indirect cost) in this patient group.

## 2 Materials and methods

### 2.1 Study design and participant recruitment

This was a cross-sectional study conducted at the Royal Prince Alfred Hospital (RPA), a quaternary hospital in Sydney, Australia from August 2022 to August 2023. Data was collected via a self-administered resource usage questionnaire developed and collected using the secure software program Research Electronic Data Capture (REDCap).

Patients over the age of 18, diagnosed with IF and receiving HPN through the hospital were recruited. Patients who had started HPN within 3 months of recruitment were excluded. All people on HPN were invited to participate via email and complete the questionnaire online. People with no email or computer access were invited to complete a paper questionnaire that was then entered into REDCap by the research team. Reminder invitations were sent at 2 and 4 weeks after the initial invitation.

### 2.2 Questionnaire design

Due to the absence of validated questionnaires to assess the patient-perceived costs associated with IF, adaptations were made from previously validated instruments measuring financial burden [16] and patient costs [17].

The questionnaire collected patient demographic information including sex, age, area of residence, education level, marital status, household size, work status, annual income and whether they had private health insurance. In this study, participants geographical location was considered regional if they were living outside a metropolitan city [18].

Participants were asked to report on all OOPE over a 12-month timeframe related to hospitalisations, general practitioner (GP) appointments, other specialist appointments (e.g. pain specialist, endocrinologist, surgeon etc.), allied health appointments (e.g. physiotherapist, psychologist, naturopath, dietitian etc.) and tests and examinations (e.g. blood tests, x-rays, iron infusions etc.). The data collection form also included distance and mode of travel, accommodation, consultation fees, equipment and society membership. Due to possible issues with recall because of multiple recurring expenses, participants were asked to recall OOPE over a 3-month timeframe related to medications (over the counter and prescription), stoma care, special foods and drinks and enteral nutrition. These expenses were then multiplied by four to estimate yearly expenses. Where participants had not been on HPN for a year, OOPE were multiplied to provide a yearly expense.

### 2.3 Data analysis

All costs are reported in AUD and rounded to the nearest dollar. OOPE for General Practitioner visits were calculated using the current Australian Medicare Benefits Scheme, AUD 41.40 reimbursed for a General Practitioner (GP) appointment lasting between 6 and 20 min [19]. All other costs were reported direct from participants’ reporting. Travel related OOPE were calculated based on mode of transportation and kilometres travelled using a cents per kilometre method [20] and other travel related costs (e.g. parking).

Current income and work status were collected. Average lost income per participant was calculated using the assumption that all participants would have been capable of working full-time in the absence of their illness. Using an average full-time income of AUD $1710.00 per week and part-time income of AUD $678 per week [21], lost income was calculated based on number of years of lost labour income for the individual since commencing HPN.

Due to small numbers, no statistical comparisons were analysed; frequencies are presented as total number and percentage, descriptive statistics are presented in the form of median and range. Observations with missing data corresponded to no expenses incurred for them.

## 3 Ethical approval

Ethical permission for the use of data was granted by the Sydney Local Health District Ethics Review Committee (RPAH Zone), Protocol No: X22–0150 & 2022/ETH00928.

## 4 Results

Thirteen participants were recruited for this survey, which was a 48 % response rate. The demographics of the participants and non-participants can be seen in Table 1. The majority of participants were female (84.6 %), residing in a metropolitan area (61.5 %) with a dysmotility diagnosis (61.5 %). The median time on HPN was 38.2 months (19.7–81.2). The median age of the participants was 35 years (26 years – 77 years) and most participants had completed Year 12 high school or above (76.9 %). The majority of participants lived with other people (76.9 %). Compared to non-participants, participants were younger, with a higher proportion of females and a motility disorder diagnosis.

**Table 1:** Participant demographics for participants and non-participants.

Demographic Characteristic	N (%) / Median (range) n = 13	Non-participants N (%) / Median (range) n = 14
Age (years)	35 (26−77)	51 (20−75)
Months on HPN	38.2 (19.7–81.2)	29 (12.0–130.1)
Mechanism of IF^a^		
Short bowel syndrome	5 (38.5 %)	7 (50 %)
Intestinal fistulae	0	0
Motility disorder	8 (61.5 %)	6 (43 %)
Extensive small bowel mucosal disease	0	1 (7 %)
Mechanical obstruction	0	0
Gender		
Male	2 (15.4 %)	5 (36 %)
Female	11 (84.6 %)	9 (64 %)
Highest education level		
Year 10 High School	3 (23.1 %)	N/C
Year 12 high school	2 (15.4 %)	N/C
Trade/certificate	3 (23.1 %)	N/C
Undergraduate university degree	2 (15.4 %)	N/C
Postgraduate university degree	2 (15.4 %)	N/C
Not applicable/other	1 (7.7 %)	N/C
Marital status		
Single	5 (38.5 %)	N/C
In a relationship	1 (7.7 %)	N/C
Married	5 (38.5 %)	N/C
Divorced	1 (7.7 %)	N/C
Widowed	1 (7.7 %)	N/C
Area of residence		
Metropolitan	8 (61.5 %)	9 (64 %)
Rural or regional^17^	5 (38.5 %)	5 (36 %)
Living situation		
Live alone	3 (23.1 %)	N/C
Live with others	10 (76.9 %)	N/C
Number of other adults	2 (1 – 4)	N/C
Number of children	1 (0 – 3)	N/C
Employment		
Unable to work due to Illness	6 (46.2 %)	N/C
Able to work	7 (53.8 %)	N/C
Casual work	1 (7.7 %)	N/C
Part-time work	3 (23.1 %)	N/C
Full-time work	1 (7.7 %)	N/C
Retired	1 (7.7 %)	N/C
Other	1 (7.7 %)	N/C
Gross annual salary range	AUD $20,000-$29,999 (<9999 – 100,000–124,999)	N/C
Financial stress caused by health		
Not at all	2 (15.4 %)	N/C
A little bit	1 (7.7 %)	N/C
Somewhat	4 (30.8 %)	N/C
Quite a bit	6 (46.2 %)	N/C
Very much	0	N/C
Private health insurance		
Yes	6 (46.2 %)	N/C
No	7 (53.8 %)	N/C

Abbreviations: HPN, home parenteral nutrition; IF, intestinal failure; N/C, not collected

a ESPEN classification of intestinal failure

### 4.1 Total out-of-pocket expenses

The median total OOPE related to health was $6099 ($3170 - $45,744) as seen in Table 2. The highest median costs were related to medications $2160 ($820 - $6000), enteral nutrition at $1340 ($0 - $3433) and other food/drink $960 ($100 - $500). Table 2 also shows OOPE based on geography. Those living in metropolitan areas spent slightly more on total OOPE across all areas of health compared to those in regional or rural areas ($6538 versus $6099 respectively). As seen in Table 3, those living in regional or rural areas spent more on travel related OOPE than those in metropolitan areas ($2587 versus $555 respectively), where highest travel related costs were related to other specialist consultations ($760 versus $210 respectively) and hospitalisation ($194 versus $121 respectively).

**Table 2:** Total out-of-pocket expenses (OOPE) based on geographic location of participant residence, presented in Australian Dollars (median and range).

Total OOPE	Metropolitan n = 8	Regional/Rural n = 5	Total n = 13
Appointments with the IF Team*	143 (64−437)	Nil	143 (64−437)
Hospitalisations*	107 (0−1691)	194 (17−2860)	107 (0−2860)
GP visits*	143 (5−2155)	17 (0–2006	77 (0−2155)
Other Specialist Consultations*	388 (86−20,279)	870 (374−2400)	388 (86−20,279)
Allied Health Consultations*	217 (69−18,174)	984 (167–1802)	217 (69−18,174)
Tests & Procedures*	71 (10−210)	68 (3−2780)	68 (3−2780)
Total Medications	2080 (820−6000)	2400 (1200−3040)	2160 (820−6000)
Over-the-counter	1152 (560−2000)	800 (720−1240)	976 (560−2000)
Prescription	1220 (0−5400)	1680 (400−3000)	1240 (0−5400)
Enteral Nutrition	1340 (0−3431)	1400 (400−2400)	1340 (0−3433)
Stoma Supplies	460 (180−600)	98 (0−560)	310 (0−600)
Other Specialised Food & Drink	960 (440−2000)	Nil	960 (440−2000)
TOTAL	6538 (3170−45,744)	6099 (3905−10,622)	6099 (3170−45,744)

Abbreviations: OOPE, out of pocket expenses; IF, intestinal failure; GP, General Practitioner

* includes costs related to travel expenses getting to and from face-to-face appointments

**Table 3:** Total travel related out-of-pocket expenses (OOPE), presented in Australian Dollars (median and range).

OOPE	Metro n = 8	Regional/Rural n = 5	Total n = 13
Appointments with the IF Team	143 (64−437)	Nil	143 (64−437)
Hospitalisations	121 (23−204)	194 (17−1260)	121 (17−1260)
GP visits	15 (0−210)	17 (2−1344)	16 (0−1344)
Other specialist Consultations	210 (38−775)	635 (374−2400)	319 (38−2400)
Allied Health Consultations	36 (14−210)	20 (17−43)	10 (0−75)
Tests & other procedures	71 (10−210)	68 (3−2780)	68 (3−2780)
Total travel related OOPE	555 (99−1354)	2587 (29.75–4498)	819 (30−1354)

### 4.2 Out of pocket expenses based on diagnosis

OOPE based on diagnosis showed that those with a motility disorder had considerably higher OOPE compared to those with other diagnoses ($7178 versus $3905), where highest costs were related to medications ($2376 versus $2000 respectively) and enteral nutrition ($1880 versus $0 respectively). Total OOPE for those with a motility disorder was $97,412 which accounts for 82.2 % of all reported OOPE.

### 4.3 Loss of labour income

The median annual reported income was $25,000; with almost one-quarter (24.4 %) of annual reported income spent on IF related costs. Six participants were unable to work due to their illness (46.2 %). Of those participants that were able to work, three had part-time employment (23.1 %) and only one participant was able to work full-time. Consequently, 84.6 % of participants reported some degree of financial stress due to their state of health, with the majority of participants reporting ‘quite a bit of stress’ (46.2 %). Average potential lost income per patient per annum was calculated as $62,310, or $1198 per week.

## 5 Discussion

This paper is the one of the first to quantify OOPE for people living with IF receiving HPN. Living with IF is not only costly to the health care system [3], [8], [9], but has high economic costs for the patient as well. Results indicate that patients spend much more than what has been reported in previous research [15], spending a median of $6099 per year on OOPE, although patient expenses were as high as $45,744 per year. Given the economic models of health care systems differ across countries and lack of research in this area, it is difficult to compare findings across services and countries. When comparing to those in the literature for different chronic diseases in Australia and internationally, the OOPE for IF are reported to be higher than most [13], [14], [22], [23], [24]. This may be due to the complexity of the patient group, with IF and HPN impacting many different body systems as well as the need for multidisciplinary supportive care [1], [2].

Health-related financial burden has been well documented across many countries and disease states [25]. Where OOPE exceed 10 % of total income, this can be considered “catastrophic” [25], [26], putting the individual at high risk of impoverishment. With a median income for this patient group of less than $30,000 per annum, the OOPE percent of this relatively small income (24.4 %) well exceeds the 10 % catastrophic threshold. As such, this already vulnerable patient group could be classified as being in catastrophic financial hardship [26]. This is also reflected in the high levels of patient-reported stress expressed by participants in this study.

Due to easy access to Medicare funded healthcare within Australia, OOPE associated with hospitalisations, clinic visits with the IF team and most tests and procedures, are at a minimum. This does not detract from other areas that are significantly higher. Of particular note and indicative of the complexity of the patient group is the high cost associated with other specialist appointments. IF services within Australia have been shown to have very limited access to specialist and supportive care [6]; and often there may be long wait-lists to see some specialist services [27]. Hence people living with IF appear to be seeking specialist services outside the public health care system culminating in high OOPE. This was particularly noticeable for those patients with a dysmotility disorder, again reflecting the wide ranging impact of IF on body systems and additional support required [1], [2], [28]. Regardless of diagnosis this finding further supports the need for extensive multidisciplinary models of care when caring for this patient group.

Enteral nutrition costs are the second highest contributor to OOPE. Where parenteral nutrition and associated ancillaries are provided at no cost to patients within the Australian health care system, access to enteral nutrition and consumables is variable across Australia [29]. While some states do provide enteral nutrition and consumables with no cost to the patient, in the state of New South Wales all costs are transferred to the patient to pay [29]. Of the 8 participants that had enteral nutrition expenses, this cost made up approximately 30 % of their total reported OOPE, which equates to 5.4 % of annual reported income. It is important to note that this patient group is relying on parenteral nutrition, and enteral nutrition is likely trophic in nature. However, due to complexity of IF, patients are likely to require more specialised (and therefore more costly) enteral feeds [30]. This additional cost of enteral nutrition and consumables may act as a deterrent for patients to move from parenteral to an enteral feeding route, as increasing the volume of enteral nutrition will have a significant financial impact for patients beyond what is reported in the results of this study. Hence further research is required to determine whether cost is a barrier for increasing enteral nutrition usage in this patient group. Despite this, discrepancies in the funding models for parenteral and enteral nutrition need to be addressed, including the feasibility of making enteral nutrition products fully subsidised in line with parenteral nutrition products.

Unsurprisingly, those in regional and rural areas spent more on travel than those living in metropolitan areas. Given the distance between their homes and specialised health care, the higher travel costs are inevitable. The introduction of virtual health care has made some specialised healthcare more accessible, as seen in the lack of travel costs required to access IF clinic appointments. However, not all health care can be delivered virtually, with many procedures and assessments requiring in-person attendance. Where possible clinical care coordination to align specialist appointments and procedures to minimise travel is important.

Interestingly, this study found that people living in metropolitan areas have greater OOPE than those in regional or rural areas. Of those living in metropolitan areas, 75 % had a dysmotility diagnosis and this group reported nearly double the number of OOPE than those without a motility disorder. Due to this anomaly further research is required to explore whether high OOPE are related to living in metropolitan areas or whether it is related to underlying disease state.

From a societal perspective, this patient group represents a major loss of workforce participation due to their illness compounded by assumed lower recreational spending. Given the indefinite nature of IF and the restricted options that allow patients to wean from HPN, such as intestinal transplantation and teduglutide, this societal (and individual) burden will continue. Research aimed at improving outcomes for this patient group, in particular quality of life and possible return to paid work are greatly lacking [4]. Designing interventions that improve outcomes and allow greater workforce involvement, contributing to individual financial benefits as well as societal should be a priority.

This results from this pilot study highlights the need for IF services to be mindful of the financial burden people living with IF may be living with and the related stress. Within the general population financial stress has been linked to lower self-esteem, increased levels of depression, insomnia, marital and parental strain, and poorer health outcomes [31]. Patients living with IF are already at high risk of the above-mentioned outcomes [32], [33]. Hence, social work involvement is vital to ensure patients are aware of and assist patients to access available benefit schemes that could lessen financial stress significantly [34]. Services should also advocate for psychological support given the widespread emotional impacts of financial stress on health and wellbeing, as well as support for loss of income and productivity.

As this was one of the first studies to examine reported OOPE in this patient group, there were limitations to the data collection, primarily focused on the unique nature of the patient group. There are currently no validated survey tools that look at the widespread direct patient OOPE in IF. The survey relied on patients reporting the costs they have incurred (i.e. recall), which despite being shown to be a valid and reliable method of data collection [35] has well documented limitations [36], [37], [38]. This study targeted a cross-section of the direct patient related costs; however, did not capture hidden or additional costs related to their illness. For example, one participant made note that they had to pay for more expensive housing in order to be able to manage HPN hygienically. The study did not ask the patients to break down their reported annual income into that from government benefit schemes versus workforce earnings. Given the indications of the major impact IF has on income from this pilot study, further multicentre research is warranted to explore a impacts over a larger cohort, addressing some of the limitations highlighted above. Specific income data including a breakdown of source of income e.g. the Disability Support Pension or National Disability Insurance Scheme should also be included. Future studies should also examine the loss of productivity for patients’ families and/or their carers due to the significant health burden.

## 6 Conclusion

This pilot study shows that there are significant OOPE for people living with IF requiring HPN, causing substantial financial stress. Patient costs are greatest in people living in metropolitan areas and those living with a dysmotility diagnosis. People living in regional areas spend more on travel. Clinicians need to ensure patients have social support to access available benefit schemes, and psychological support to manage the emotional impacts of financial stress. More research is urgently needed to further understand the extent and impact of the economic burden of IF from the patient and broader societal perspectives.

## CRediT authorship contribution statement

**Quiney Lin:** Writing – review & editing, Investigation, Formal analysis. **Liz Beyer:** Writing – review & editing, Investigation, Data curation. **Michelle Cunich:** Writing – review & editing, Methodology, Formal analysis. **Sharon Carey:** Writing – review & editing, Writing – original draft, Supervision, Project administration, Methodology, Investigation, Formal analysis, Conceptualization. **Maddison Breen:** Writing – review & editing, Writing – original draft, Formal analysis.

## Consent

All participants consented to be included in this study.

## Ethical clearance

Full ethics details included in body of the paper

## Funding

This research did not receive any specific grant from funding agencies in the public, commercial or not-for-profit sectors.

## Declaration of Competing Interest

None
